# A systematic survey to identify lethal recessive variation in highly managed pig populations

**DOI:** 10.1186/s12864-017-4278-1

**Published:** 2017-11-09

**Authors:** Martijn F. L. Derks, Hendrik-Jan Megens, Mirte Bosse, Marcos S. Lopes, Barbara Harlizius, Martien A. M. Groenen

**Affiliations:** 1 Wageningen University & Research, Animal Breeding and Genomics, Wageningen, The Netherlands; 2 Topigs Norsvin Research Center, Beuningen, the Netherlands; 3 Topigs Norsvin, Curitiba, Brazil

**Keywords:** Population genomics, Genetics, Deleterious variation, Embryonic lethality, Mummified piglets

## Abstract

**Background:**

Lethal recessive variation can cause prenatal death of homozygous offspring. Although usually present at low-frequency in populations, the impact on individual fitness can be substantial. Until recently, the presence of recessive embryonic lethal variation could only be measured indirectly through reduced fertility. In this study, we estimate the presence of genetic loci associated with both early and late termination of development during gestation in pigs from the wealth of genome data routinely generated by a commercial breeding company.

**Results:**

We examined three commercial pig (*Sus scrofa)* populations for potentially deleterious genetic variation based on 80 K SNP-chip genotypes, and estimate the effects on reproductive traits. 24,000 pigs from three populations were analyzed for missing or depletion of homozygous haplotypes. We identified 145 haplotypes (ranging from 0.5–4 Mb in size) in the genome with complete absence or depletion of homozygous animals. Thirty-five haplotypes show a negative effect on at least one of the analysed reproductive traits (total number born, number of stillborn, and number of mummified piglets). One variant in particular appeared to result in relative late termination of development of fetuses, responsible for a significant fraction of observed stillborn piglets (‘mummies’), as they die mid-gestation. Moreover, we identified the *BMPER* gene as a likely candidate underlying this phenomenon.

**Conclusions:**

Our study shows that although lethal recessive variation is present, the frequency of these alleles is invariably low in these highly managed populations. Nevertheless, due to cumulative effects of deleterious variants, large numbers of affected offspring are produced. Furthermore, our study demonstrates the use of a large-scale commercial genetic experiment to systematically screen for ‘natural knockouts’ that can increase understanding of gene function.

**Electronic supplementary material:**

The online version of this article (10.1186/s12864-017-4278-1) contains supplementary material, which is available to authorized users.

## Background

Small effective population size can lead to inbreeding depression. The cause of inbreeding depression is the accumulation of (recessive) deleterious alleles increasing in frequency and becoming expressed in homozygous state due to drift in small populations [1]. In domesticated populations, despite strong artificial selection for desired traits, selection on relatively rare variation (allele frequency < 10%) is usually very inefficient [2]. The eradication of deleterious variation is challenging by applying either traditional or even more recent ‘genomic’ breeding strategies. The inefficiency of purging deleterious variation, even from highly managed populations, is particularly apparent if there is an unpredictable, or poorly characterized relationship between genotype and phenotype. For instance, when a homozygous deleterious phenotype leads to very early death of the developing embryo, the only observed consequence is a (somewhat) lower fertility of the parents. It is estimated that livestock species harbour 2–4 fold higher variation in the genome compared to humans [3, 4]. Accordingly, the number of non-synonymous mutations has been shown to be greater in livestock animals, suggesting a large reservoir of potentially deleterious variation in livestock [3].

Domestication in general, and modern breeding industry in particular, constitutes the largest and longest lasting genetic experiment ever conducted. In many cases, especially in pig and poultry breeding, commercial breeding organizations apply their genetic improvement efforts at a small number of elite breeding lines [5]. These breeding lines are usually fairly closed, i.e. exchange between breeding lines is infrequent. Moreover, depending on species and breeding purpose, the effective populations sizes vary from small (several hundred at most) to very small (dozens of animals). With the adoption of genomic selection, a large proportion of the animals in the pure bred elite lines, i.e. the selection candidates, are genotyped using high to medium density SNP assays to estimate their genomic estimated breeding value (GEBV). Breeding values itself are not very efficient in eliminating rare deleterious variation. However, the availability of a large number of genotyped, pedigreed individuals enables the unravelling of the genetic basis of rare disorders in the population. Recessive deleterious variants can be identified by testing for statistical depletion, or even the absence, of specific haplotypes in homozygous state. This haplotype approach is a powerful tool [6–10] originally developed for cattle by vanRaden et al. (2011). The power of this method heavily depends on the number of genotyped individuals. If applied to tens of thousands of genotyped animals, a number prohibitively large usually for academic budget, but routinely attained in many commercial breeding lines nowadays, even very rare deleterious haplotypes can be detected (frequency < 2%). Genotyping large numbers of domestic animals currently still relies on the use of dedicated SNP assays (‘SNP chips’). Since these assays are designed with a bias towards high minor allele frequency (MAF), causal variants of serious syndromes are unlikely to be present on the assay. The haplotype based approach is therefore more efficient in capturing deleterious variation compared to individual SNPs, which are expected to be often in low linkage disequilibrium (LD) with the causal variant. Significant depletion of haplotype homozygosity is an indication of decreased viability, and these haplotypes are likely to harbour deleterious mutations causing embryonic lethality (EL) in homozygous state. Studies in mouse showed a lethal knockout phenotype for about 30% of mouse genes in homozygous state, suggesting a large proportion of potential embryonic lethal genes in Mammalia [11].

Fertility traits, such as total number born (TNB), are among the relevant phenotypes systematically recorded in pig breeding that can provide phenotypic support for (early) embryonic lethality. In addition, recorded phenotypes, such as number of stillborn (NSB) and the number of mummified pigs (MUM), can provide additional information on genetic defects that are lethal, or seriously compromising the survival probability later in foetal development. In theory, assuming prenatal death of homozygotes, a loss of 25% of each litter is expected if two heterozygous carriers of the lethal variant mate (C x C mating). In mammals, death of an embryo or foetus usually does not result in spontaneous termination of the pregnancy, when also carrying living young. Instead, foetuses go through a process of desiccation and encapsulation, known as mummification [12]. Foetal mummification can occur from day 35 of gestation until parturition (when the skeletal system is developing), and has been associated with several risk factors like large litter size and infectious disease [13]. Also, several studies have already identified QTLs associated with pig reproductive traits, including mummified piglets [14–17]. Parameters, such as time of death and any morphological abnormalities found in mummified piglets [12], can provide insights in the developmental consequences of a specific genetic defect without any further welfare concerns for the mother or live siblings. A systematic analysis to identify specific genetic defects as risk factors, however, has not been conducted.

In this study, we aim to identify novel genetic loci associated with both early and late termination of development during gestation in pigs. Moreover, we examine the occurrence and frequency of lethal haplotypes in the studied breeds, as well as their impact on fertility related traits. Finally, we show that missing homozygosity in highly managed livestock populations can be a result of early lethality caused by low frequency recessive lethal haplotypes.

## Results

### Screening for haplotypes exhibiting missing or deficit homozygosity

Breeding lines are used in three- or four way crosses to produce large numbers of slaughter pigs [5]. However, elite breeding lines are generally kept as closed populations, and selection is done within these populations. Because of this characteristic, these breeding lines meet two criteria for the method applied in this study to be successful: a) we can expect that not all deleterious variation is effectively purged from the population, and that low to moderate allele frequencies for some deleterious variation remains in the population, and b) because we specifically examine C x C matings, we expect 25% of the offspring to be homozygous for the carrier haplotype, a necessary prerequisite when scanning for missing homozygotes. In total, we scanned for missing homozygosity in 5517 pigs from a synthetic elite sire (BR) line with Large White and Piétrain genetic background, 5301 Landrace (LR) and 12,982 Large White (LW) pigs, the latter two representing two elite dam lines. The dam and sire lines are selected towards distinct breeding goals. Dam populations are primarily selected for female reproductive traits, whereas sire lines are primarily selected for production traits. We found no evidence of (recent) inbreeding in any of the three lines (F-coefficient close to zero, Additional file 1: Figure S1). The statistical power of our study stems from a total of 23,800 animals from the three pure lines, genotyped on low to medium (10 K, 60 K, and 80 K) density SNP arrays (Additional file 1: Table S1-S2). Animals genotyped on either the 10 K or 60 K panel were imputed to 80 K with generally high accuracies (Additional file 1: Table S3). After filtering, a final set of 22,961 animals was used for further analysis (Additional file 1: Table S4). After haplotype phasing, we systematically examined the genome by using an overlapping sliding window approach to assess haplotype frequencies. Haplotypes were marked as potentially deleterious if a significant deficit (exact binomial test) in homozygotes was observed.

We identified 22, 10, and 56 haplotypes with missing homozygosity (MH), and 19, 6, 32 haplotypes exhibiting a statistically significant deficit homozygosity (DH) in the BR, LR, and LW line, respectively (Table 1, Additional file 1: Figures S2-S4**,** Additional file 2). DH haplotypes have either incomplete LD with the causal variant or incomplete penetrance of the variant at the phenotypic level in homozygous state. The haplotype lengths varies from 0.5 to 4 Mb and frequencies range from 0.8 to 11.4% for haplotypes with MH and from 2.6 to 15.8% for haplotypes with DH. The larger number of genotyped animals and trios (both parents and offspring genotyped) in the LW breed allowed for the identification of a high number of low frequency haplotypes (< 3%), compared to the other two breeds (Additional file 1: Figure S5). For the haplotypes with significant depleted homozygosity, the number of expected homozygotes ranged from 5.75 to 140.25 animals per haplotype, with an overall average of 18.4, 22.2, and 24.3 expected homozygotes for LR, LW, and BR breeds, respectively (Additional file 1: Figure S6). We expect a larger proportion of heterozygous carriers from C x C litters due to the missing homozygote offspring. Hence, the percentage of heterozygous carrier offspring is greater than 50% for the majority of the haplotypes in all three breeds (Table 1 **,** Additional file 2).

**Table 1:** Description of the data for missing and depleted homozygous haplotypes in three pig breeds. Table shows average and standard deviation (between parenthesis) for all parameters per breed. The number of loci harbours the unique number of genomic windows containing significant haplotypes

Description	Synthetic boar line	Landrace	Large white
Number of samples	5488	5056	12,417
Number of trios	3806	2548	8778
Number of haplotypes	41	16	88
Number of loci	32	16	70
Haplotype length (markers)	25.4 (18.5)	19.3 (22.3)	36.04 (37.0)
Haplotypes in window (frequency > 0.5%)	16.3 (7.8)	22.2 (10.3)	25.1 (13.3)
Number of carriers	707.5 (348.9)	689.9 (290.8)	972.1 (545.3)
Haplotype frequency	6.4 (3.2)	6.8 (2.9)	3.9 (2.2)
Homozygous expected	24.3 (27.6)	18.4 (10.5)	22.2 (22.6)
Carrier matings with genotyped offspring	29.9 (31.6)	36.1 (20.6)	33.6 (34.6)
Carrier matings in pedigree	72.9 (69.5)	169.8 (119.0)	104.7 (107.8)
Genotyped carrier progeny	96.8 (109.7)	73.75 (42.1)	88.8 (90.2)
% Heterozygous carrier progeny	55.1	60.3	53.8
Genes in window	21.2 (26.3)	15.8 (22.0)	19.0 (19.7)

### Genomic regions enriched for missing or deficit homozygosity

We identified four regions enriched for MH and DH haplotypes over all three breeds: SSC1:294–297.5, SSC2:156–159.75, SSC3:142–144, and SSC11:70–73.5 Mb (Additional file 3). Together these four regions account for 42 of the total of 145 identified haplotypes identified in all three lines. These loci have previously been identified as copy number variable regions [18], but were not extensively linked to reproductive traits [19]. A gene-set enrichment analysis for the genes overlapping the 145 identified haplotypes revealed only one significant annotation cluster, i.e. related to olfactory receptors (DAVID enrichment score 3.45) (Additional file 3, Table S5).

### Association with reproductive traits and candidate gene selection

**Fig. 1:**
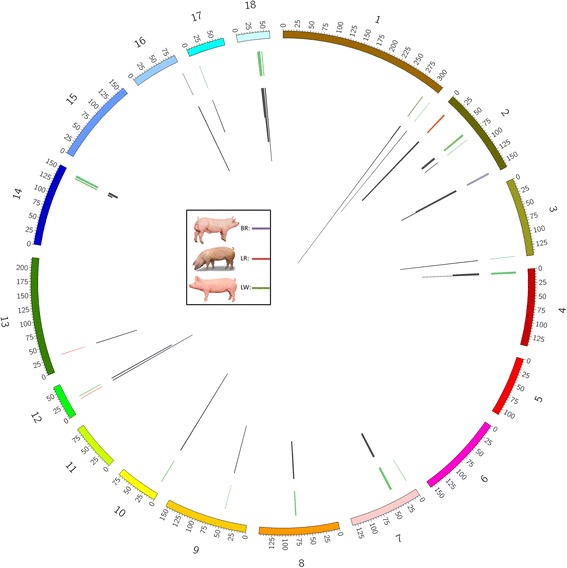
Genomic locations of the haplotypes affecting fertility in the BR (*purple*), LR (*red*), and LW (*green*) breed. Figure shows 18 autosomal chromosomes, line width indicates haplotype length. Black lines indicate the relative haplotype frequency ranging from 1.0 to 11.5%. Pig graphics in the figure legend provided by Topigs-Norsvin, all rights reserved

We examined all 145 significant haplotypes for their effect on three reproductive traits: TNB, NSB, and MUM. Phenotypic records for all three traits were available for both dam lines. For the boar line, only records on TNB were available. We listed all phenotypes from C x C matings and carrier x non-carrier matings (C x NC) to identify missing or depleted haplotypes affecting these traits. Haplotypes significantly affecting fertility are named and ranked according to breed, affected phenotype, and genomic location. Figure 1 shows the genomic distribution of the haplotypes affecting fertility per breed.

#### Total number born

We identified 26 haplotypes exhibiting a significant reduction in TNB (Table 2), three in the BR line, 4 in LR, and 19 in LW. The reduction in TNB ranged from 2.84 to 18.72% representing 0.45 to 2.97 piglets per litter. Candidate genes were identified based on early lethality in knockout mice studies and could be identified for 16 haplotypes (Additional file 4). Fourteen regions were not previously associated with reproductive traits in livestock and can be considered as novel according to the 2016 pig QTL database [19] (Additional file 4). Six haplotypes exhibit a large reduction (> 10%) of TNB. LR4, found on SSC13, shows a reduction of 17.13% in TNB based on 44 C x C matings. This 0.5 Mb region contains 12 protein coding genes (Additional file 4), of which *KLHL40* and *POMGNT2* cause early lethality in knockout mice [20]. Moreover, haplotype LW9, spanning a 4 Mb region on SSC7, exhibits a reduction in TNB of 15.61% and overlaps with 13 candidate genes that could potentially cause early lethality [20]. Haplotype LW14, previously associated with TNB [15], shows a reduction of 11.25% in TNB. This region contains two candidate genes, one causing early embryonic lethality before time of implantation in mice (*UROS*), and the other causing post-natal lethality (*ADAM12*) [20]. Finally, four haplotypes were identified on SSC18, of which LW19 spans a 1 Mb region (43–44 Mb) and exhibits the largest reduction on TNB (18.72%) based on 88 C x C matings. Moreover, this haplotype has been associated with a large increase in the number of mummified piglets and a strong candidate gene could be identified (*BMPER* described below: *LW19 homozygous foetuses become mummified in Large White*).

**Table 2:** Haplotypes affecting TNB. The genomic location and haplotype frequency is provided in columns 1–5. The “homozygotes” section shows expected and observed homozygotes including statistical test. Information on carrier x carrier (C x C) matings and progeny is provided in the “matings” section. Effect on the phenotype is provided in the “reduction in TNB” section

Abbreviation	Chr	Start	End	Hap. Freq	Homozygotes			Matings			Reduction in TNB	
					Expected	Observed	Exact binomial test	C x C matings	Genotyped progeny	Het. carrier progeny	Percent	P
BR1	SSC2	156	159	3.8	8	0	0.000138	42	32	13	8.192	0.054
BR2	SSC2	158.5	159.5	4.8	18.75	0	5.70E-10	69	75	38	9.220	0.008
BR3	SSC16	85.5	86.5	5.4	17.25	1	8.22E-08	53	69	23	7.037	0.046
LR1	SSC1	295	296	11.4	30.25	0	1.71E-15	370	121	97	3.243	0.012
LR2	SSC2	10.5	13.5	6	6.75	0	0.000585	148	27	18	4.35	0.026
LR3	SSC8	78	79	2.6	8.25	0	0.000144	30	33	17	8.505	0.017
LR4	SSC13	28.75	29.25	3.6	5.75	0	0.002581	44	23	16	17.130	2.16E-06
LW1	SSC1	294.5	295.5	2	7.5	0	0.000138	44	30	20	9.552	0.010
LW2	SSC1	294.75	295.25	11.5	132.75	1	1.87E-72	619	531	418	2.843	0.007
LW3	SSC1	295	295.5	5.9	30.25	0	5.67E-17	259	121	94	4.522	0.003
LW4	SSC1	295	295.5	5.1	37.5	2	1.16E-19	206	150	116	4.213	0.008
LW5	SSC1	313.75	314.25	7.4	126.75	3	3.00E-60	483	507	146	3.875	0.000121
LW6	SSC3	142.75	143.25	5.8	24.25	0	4.27E-14	207	97	31	4.275	0.012
LW7	SSC4	4	8	2.6	26.25	5	7.04E-10	111	105	45	5.311	0.013
LW8	SSC4	6.5	7.5	4.5	14.25	1	4.16E-08	50	57	32	9.854	0.009
LW9	SSC7	40	44	2.6	6.75	0	0.000138	30	27	12	15.612	0.001
LW10	SSC8	78	80	3.3	19.5	5	5.44E-09	173	78	60	4.200	0.023
LW11	SSC9	46.75	47.25	4	6	0	0.00036	56	24	16	7.622	0.012
LW12	SSC10	5.5	6.5	6.5	50.25	5	9.23E-23	296	201	123	4.169	0.003
LW13	SSC12	26.5	27.5	5.2	20	0	9.31E-11	102	80	52	7.383	0.004
LW14	SSC14	146	150	1.6	8	0	0.000144	28	32	15	11.252	0.004
LW15	SSC17	10.5	11	3.1	16	0	3.80E-09	42	64	29	9.920	0.012
LW16	SSC18	34.5	37.5	2.8	6	0	0.000954	30	24	14	11.621	0.027
LW17	SSC18	36	40	4.3	15.25	2	5.22E-07	74	61	28	7.517	0.017
LW18	SSC18	42.75	43.25	5.5	15.75	3	1.32E-07	127	63	43	15.438	1.27E-12
LW19	SSC18	43	44	4.3	11.75	0	1.29E-06	88	47	32	18.715	4.91E-12

#### Number of stillborn

Four haplotypes with a significant increase in the number of stillborn were identified (Table 3 **)**. The increase ranged from 29.8 to 57.7%, representing an increase of 0.42 to 0.97 stillbirths per litter. Candidate genes could be assigned to each haplotype (Additional file 4), but none of the haplotypes has previously been associated with an increased number of stillbirths in livestock according to the 2016 pig QTL database [19].

**Table 3:** Haplotypes affecting NSB. The genomic location and haplotype frequency is provided in columns 1–5. The “homozygotes” section shows expected and observed homozygotes including statistical test. Information on carrier x carrier (C x C) matings and progeny is provided in the “matings” section. Effect on the phenotype is provided in the “Increase in stillborn” section

Abbreviation	Chr	Start	End	Hap. Freq	Homozygotes			Matings			Increase in stillborn	
					Expected	Observed	Exact binomial test	C x C matings	Genotyped progeny	Het. carrier progeny	Percent	P
LR5	SSC12	21	21.5	6.6	20	0	1.37E-10	189	80	63	29.787	0.016
LW20	SSC2	64	68	2	9.25	0	2.03E-05	160	37	24	32.215	0.007
LW21	SSC2	78.25	78.75	2	8.5	0	5.35E-05	165	34	21	34.228	0.004
LW22	SSC14	142	144	1.3	8.5	0	8.53E-05	20	34	15	57.738	0.038

#### Number of mummified piglets

Analysis of the number of mummified piglets revealed five haplotypes with a significant increase in mummified piglets per litter (Table 4). The increase ranged from 37.1 to 479.4%, accounting for an increase of 0.16 to 1.64 mummified piglets per litter for C x C matings compared to C x NC matings. Also, two haplotypes (LW17, LW23) were found in a region previously associated with other reproductive traits [19]. One of these, LW23, located on SSC7 (6.75–7.25 Mb), shows a 2.5 fold increase in MUM, but no candidate gene could be assigned (Additional file 4). Finally, three haplotypes were identified on SSC18, one of these, LW19, exhibits a complete lack of homozygotes, and shows the largest increase (about 5-fold) in the number of mummified piglets. The two additional haplotypes on SSC18 surrounding LW19 (LW17, LW18), exhibit similar, but less severe, phenotypic effects. We observed a low number of homozygous carriers for LW17 (2 homozygotes) and LW18 (3 homozygotes), suggesting incomplete LD with the causal variant.

**Table 4:** Haplotypes affecting MUM. The genomic location and haplotype frequency is provided in columns 1–5. The “homozygotes” section shows expected and observed homozygotes including statistical test. Information on carrier x carrier (C x C) matings and progeny is provided in the “matings” section. Effect on the phenotype is provided in the “Increase in mummified” section. Haplotype already listed in Table 2 have similar abbreviations

Abbreviation	Chr	Start	End	Hap. Freq.	Homozygotes			Matings			Increase in mummified	
					Expected	Observed	Exact binomial test	C x C matings	Genotyped progeny	Het. carrier progeny	Percent	P
LW4	SSC1	295	295.5	5.1	37.5	2	3.05E-19	206	150	116	37.209	0.012
LW23	SSC7	6.75	7.25	1	7.25	0	0.000954	17	29	13	256.522	0.028
LW17	SSC18	36	40	4.3	6	2	5.22E-07	30	24	14	224.324	0.044
LW18	SSC18	42.75	43.25	5.5	15.75	3	1.32E-07	127	63	43	375.757	1.67E-10
LW19	SSC18	43	44	4.3	11.75	0	1.29E-06	88	47	32	479.412	2.118E-10

### LW19 homozygous foetuses become mummified in large white

Haplotype LW19 (SSC18:43–44 Mb) shows a five-fold increase in the number of mummified piglets, and a 18.71% decrease in TNB calculated from 88 C x C matings (Table 5). This locus has not been previously reported to be associated with an increase in the number of mummified piglets.

**Table 5:** Haplotype LW19 characteristics. Difference is the percentual difference in the average TNB and MUM for C x C and C x NC matings

Haplotype ID	LW19
Position, Mb	SSC18: 43–44
Number of markers	26
Starting marker	ASGA0079708
Ending marker	ALGA0098146
Haplotype frequency %	4.3
Carrier frequency %	8.6
Avg. TNB (difference %)	12.9 (−18.7)
Avg. NBA (difference %)	11.89 (−17.7)
Avg. Mummified (difference %)	1.97 (479.4)
Genes in window	BMPER, BBS9

**Fig. 2:**
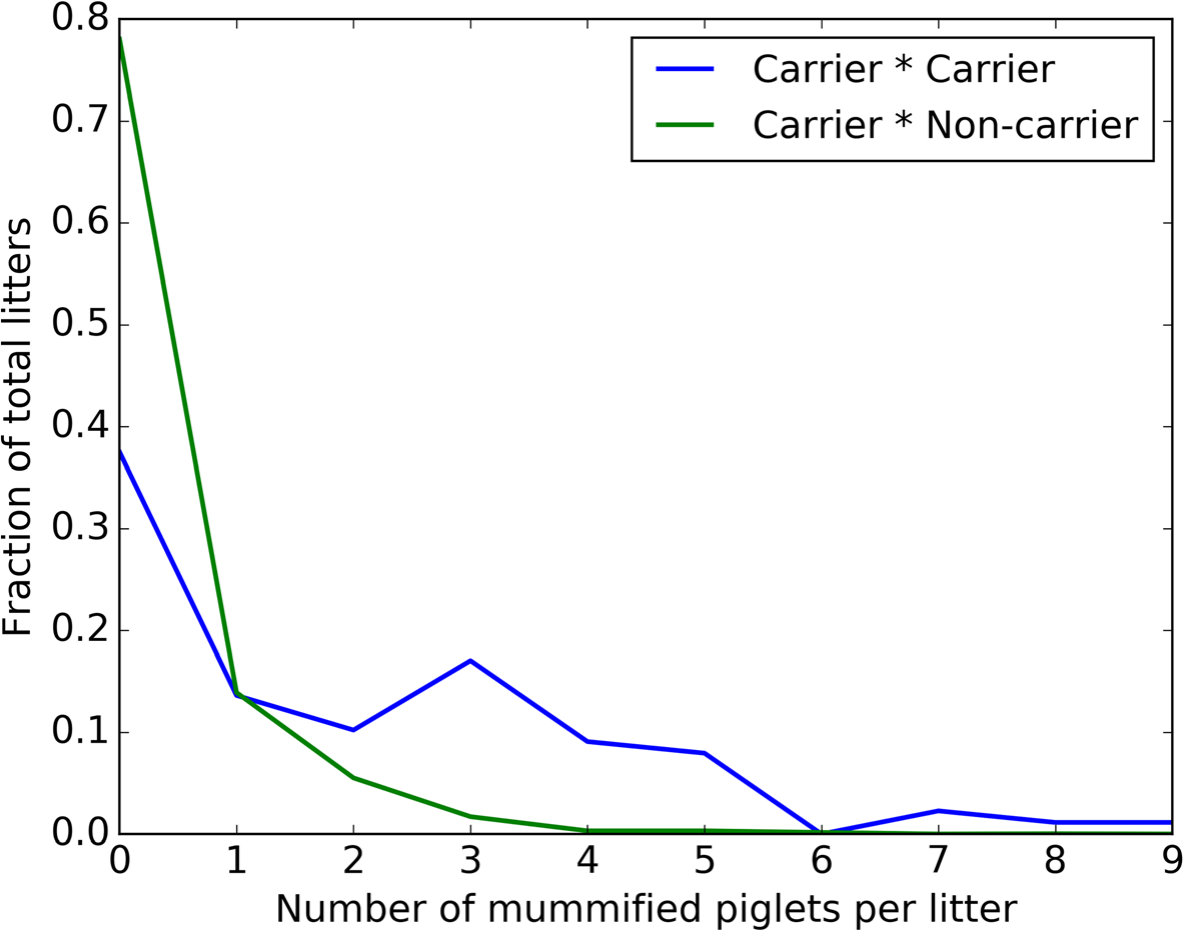
Fraction of the number of mummified piglets per litter for haplotype LW19. The axes indicate the fraction of the total litters (y) with a certain number of mummified piglets (x). Figure shows a larger proportion of mummified piglets per litter for the C x C matings compared to C x NC matings, except when *n* = 1

Together these 88 matings produced 173 mummified piglets, 1.97 on average per litter (Table 5 **,** Additional file 1 **:** Figures S7-S8). Moreover these 173 mummified piglets are responsible for 1.98% of the total number of mummified piglets (8726) recorded for this breed in a decade (December 2006–April 2016). The difference in the ratio MUM/NSB/NBA between C x C (1.97/1.01/11.89) compared to C x NC (0.34/1.43/14.44) is highly significant (*P* < .0001, Chi-Square). Especially the fraction of litters that contain 2 to 5 mummified piglets per litter is significantly higher for C x C matings (Fig. 2).

**Fig. 3:**
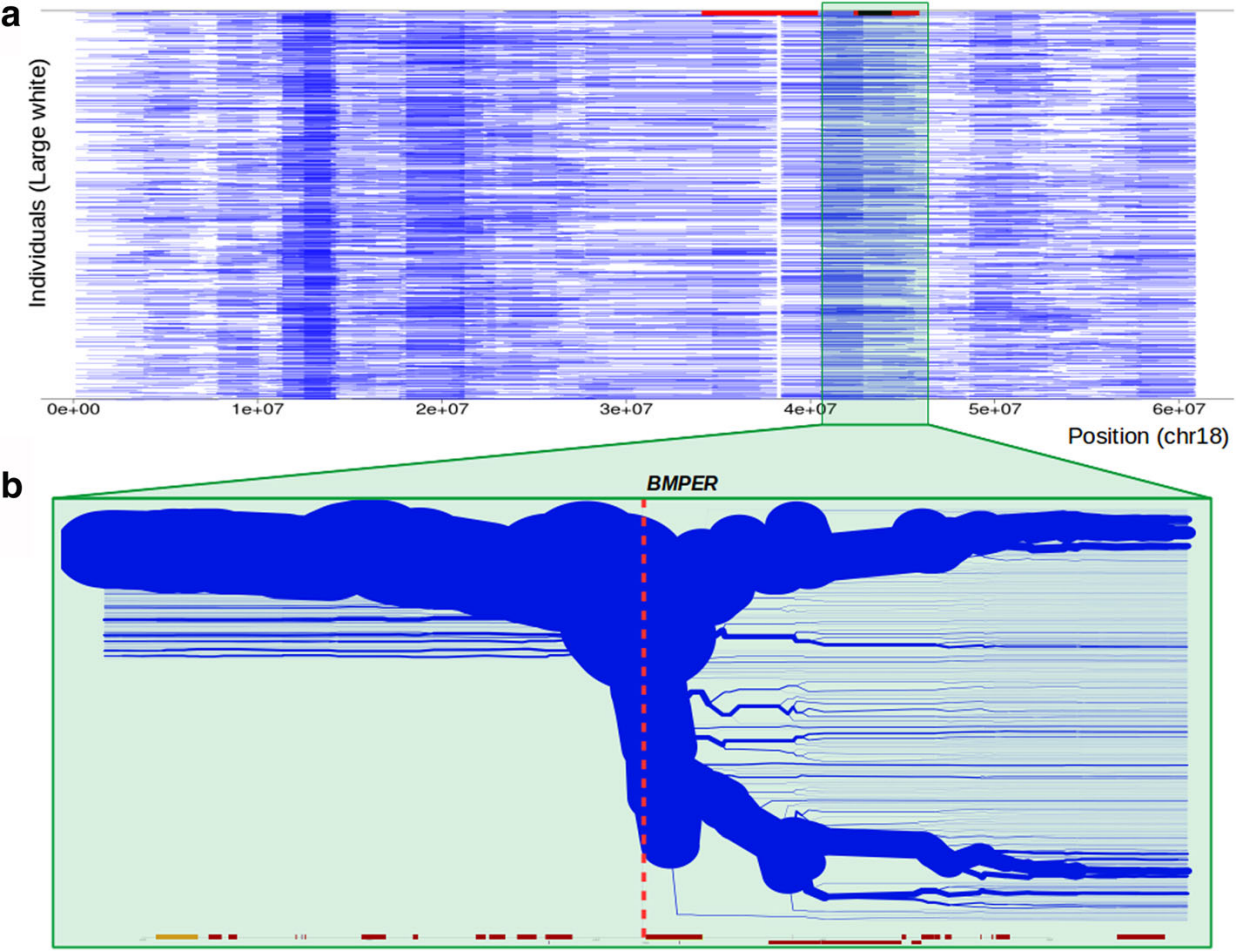
Runs of homozygosity (ROH) and extended haplotype homozygosity (EHH) on SSC18. **a** Individual Large White pigs are represented as horizontal lines, with *blue bars* indicating a homozygous segment at that position on SSC18. The *red bars* on top indicate all significant haplotypes in the Large White, with the haplotype LW19 (SSC18:43-44 Mb) indicated in *black*. Clustered homozygous segments are an indication of a haplotype putative under selection. **b** Local breakdown of LD in the Large White population at the LW19 haplotype locus. The bifurcation diagram displays haplotypes starting at the BMPER locus and extending either up- or downstream of the BMPER gene. Line thickness represents proportion of haplotypes. *Red* and *yellow bars* indicate locations of genes as annotated in Ensembl (release 87)

The carrier frequency for this haplotype is 8.6%, meaning that about 0.74% of the litters in this breed are affected assuming random matings, and 0.185% of all piglets will be affected if penetrance is 100%. We tracked three recent C x C matings with a total of 9 mummified piglets to estimate the approximate age when the foetus has died (an example of a C x C mummified piglet is shown in Additional file 1: Figure S9**).** The length from crown to rump was about 10–11 cm which corresponds to an age of approximately 55 days [21]. The haplotype overlaps with two protein coding genes (*BMPER, BBS9*). BBS9 has previously been associated with the Bardet-Biedl syndrome in human. This syndrome, however, does not usually cause early lethality [22]*.* We therefore focused on the *BMPER* gene as the most likely candidate gene for the observed effect. We performed runs of homozygosity (ROH) and extended haplotype homozygosity (EHH) analysis on SSC18, and identified a region flanking the BMPER locus to be potentially under recent positive selection (SSC18:40–43 Mb, Fig. 3).

## Discussion

Highly managed, domesticated populations are expected to be under selection against inbreeding depression. Indeed, our results show that high frequency occurrence of potentially monogenic lethal or debilitating alleles is rare in commercial populations, despite relatively low effective population sizes. This is in contrast to other domesticated populations that are far less well managed, such as dogs and horses, and that can carry high frequencies of deleterious alleles [23, 24]. The few examples that exist for commercial breeding populations, mostly from Holstein cattle, indicate that the effects of deleterious traits are often masked, because they involve early embryonic lethality, which reveals itself only indirectly as depressed parent fertility. Moreover, some of these lethal alleles are maintained in the population as a result of balancing selection, where heterozygotes show an advantageous phenotype [25]. However, even in those cases, these alleles are usually kept at low frequency.

It is unlikely that purging can remove all or even most of the detrimental variation because even modern genomic breeding programs are inefficient in capturing genotype-phenotype relations of low frequency alleles. Our study reveals that the frequency of the haplotypes exhibiting missing homozygosity ranges from 0.5–11%, showing that we have the statistical power to detect very rare deleterious haplotypes in our populations, but also confirming that, as expected, truly lethal recessive variants are invariably infrequent.

The approach chosen for this study relies on the premise that unexpected absence of homozygotes results from unviability of the homozygous deleterious allelic state. Ideally, if we would have sequence data for many thousands of animals, we would be able to directly infer the absence of specific homozygous allele states, e.g. alleles that impair the required protein function, or, alternatively, alleles that are in complete LD with such variants. However, we used low to medium density genotype data, and the SNPs on the chips have been primarily chosen based on their relatively high MAF in most breeds, unlikely to include deleterious variants. To overcome this problem, the haplotype based analysis chosen in this study applied a sliding window approach from 0.5 to 4 Mb. Selecting optimum window sizes is not trivial and depends on population structure, SNP density, recombination rate, and haplotype frequencies within the examined genomic region. For example, by selecting large haplotypes, we increase the risk of analysing recombinant haplotypes. Moreover, by selecting very small haplotypes, non-unique haplotypes might be selected (overlap between distinct haplotypes). We solely used information from complete trios (both parents and offspring genotyped) to calculate the expected number of homozygotes. This number, however, is likely an underestimation, because not all genotyped animals are in genotyped trios. Also, the haplotype approach is unlikely to capture all deleterious variants, as any rare variant that resides only on a common haplotype will be missed. However, rare variants that coincide with rare haplotypes can be robustly detected, with the number of genotyped offspring being the limiting factor for statistical power.

In total, 145 haplotypes showed a significant deficit of homozygotes. Of these, 35 haplotypes showed a negative effect on at least one of the three fertility traits examined, indicating that indeed these 145 haplotypes are highly enriched for variation that can lead to embryonic lethality or prenatal death. The overwhelming majority of these haplotypes are located at chromosomal regions not previously linked to fertility [19]. Only four genomic loci are shared between the three breeding populations, and all four of these were previously identified as copy number variable (CNV) regions [18]. We hypothesize that these four haplotypes are likely false positives, as these CNV events can cause duplication of genetic markers potentially introducing polymorphisms. This could lead to inter-locus cross-hybridization of oligo’s on the chip, causing all individuals to become heterozygous for a particular marker, or a set of markers, generating haplotypes with missing homozygotes.

Several of the identified haplotypes did not show a significant effect on fertility. In some cases, the number of C x C matings was too low to obtain significant statistical support. There are three additional explanations for the absence of an effect on fertility. First, recombination hotspots can potentially result in an excess of heterozygotes that carry recombinant haplotypes. This seems especially apparent at the chromosome ends. Second, since we are examining commercial breeding lines, there is selection on the animals that are genotyped. Piglets are selected for genotyping based on their performance on numerous traits depending on their particular breeding purpose, e.g. growth rate, back fat, fertility, number of teats, and leg quality. Piglets that exhibit unfavourable phenotypes early in life are likely not genotyped and could end up as “missing homozygotes” in our analysis. One example of such a phenotype in pig breeding is the number of teats. Piglets with less than 12 teats are often immediately removed from the population. Third, embryos dying very early (e.g. before time of implantation) are likely replaced by other embryos, as breeding sows are very likely to produce a far higher number of ova than can be accommodated in the uterus.

For one specific haplotype (LW19), identified only in the Large White line, we found evidence from 88 carrier matings that homozygous animals die mid-gestation, and become mummified (fivefold increase in the number of mummified piglets). The association of mummified piglets and haplotype LW19 is very likely an underestimation, since the number of mummified piglets is not always recorded equally strict at all breeding farms, especially for the embryos that died early in gestation for which mummies are small. The haplotype showed a 19% reduction in TNB, less than the 25% loss expected under HWE. One explanation for this discrepancy is that the whole litter might potentially be aborted if a large proportion of the litter dies during gestation, and will therefore not be recorded. We identified the *BMPER* gene as a likely candidate gene causing the defect. In human, the perinatal lethal skeletal disorder diaphanospondylodysostosis (OMIM: 608,022) is associated with homozygous or compound heterozygous mutations in the *BMPER* gene [26]. Characteristics include a small chest, abnormal vertebral segmentation, and posterior rib gaps [26]. Homozygous knockout mice exhibit neonatal lethality associated with abnormal lung and skeleton development [27, 28]. Moreover, heterozygotes for a null allele exhibit abnormal lung development [28]. *BMPER* is involved in the negative regulation of bone morphogenetic proteins (BMPs), a group of growth factors involved in the formation of bone and cartilage [29]. Variation in this gene has been associated with increased body size and rump length in cattle [30], and higher intramuscular fat content in pig [31]. Evidence for similar early termination of development comes from human and mouse studies [26, 28]. We observed multiple haplotypes with a deficit of homozygotes surrounding the LW19 haplotype associated with similar phenotypic effects (increase in number of mummified piglets, decrease in TNB). It is likely that these haplotypes are not in complete LD with the causal variant. Interestingly, these haplotypes are surrounding a region under selection, despite low LD with the selected haplotype, we hypothesize that this could be a remnant of genetic hitchhiking in the past as this locus has previously been associated with increased body weight and ovulation rate [17, 32]. Therefore, LW19 might have been subjected to genetic hitchhiking, although we did not find direct evidence supporting this (LW19 is not in LD with the neighbouring haplotypes under selection). More recent recombination might have lowered the LD, but as a result of previous hitchhiking the haplotype still segregates in the population.

Despite the limited impact on crossbred products, given that most haplotypes are population specific, eradication of these haplotypes is still desired. Especially because embryonic lethality leading to mummification does not only have negative economic consequences for the pig breeder, but also results in reduced animal welfare such as health risk for the sow [12]. Our study can directly impact positively on current breeding programmes, by avoiding C x C matings to lower the frequency of the lethal recessive haplotypes in the elite breeding lines. Furthermore, if a causal variant is found, avoiding homozygotes could be combined with a low-level selection to eradicate the variant after a number of generations. Many risk factors have been associated with an increase in mummified piglets [33], most of them, however, are independent of the foetus’s genetic material. In our study, we found that about 2% of all recorded mummies in the Large White breed can be attributed to C x C matings for the LW19 haplotype. We believe that the majority of the total recorded mummified piglets are not a direct effect of a genetic defect carried by the unborn foetus, because several other factors, especially many pathogens can cause mummified or stillborn piglets. Therefore, this proportion of 2% likely represents a much larger fraction of the total number of mummified piglets directly caused by a genetic defect carried by the unborn foetus.

The use of molecular tools, in particular in genomic selection, has considerably increased breeding progress over the past years. Despite this, identification of low-frequency deleterious recessive alleles, present in livestock populations, remains a challenging task. The expected low frequency of this type of variation, and therefore marginal effects on fertility traits at the population, contribute to this challenge. However, the routine large scale genotyping of domesticated animals has opened up new possibilities to detect these low frequency deleterious alleles. A systematic genomic survey for missing homozygosity, as applied in this study, is especially promising when thousands of individuals are genotyped. Using this method, novel genetic defects can be identified and fully characterised. Moreover, the increased use of whole genome sequence (WGS) data for breeding purposes opens new opportunities to directly infer deleterious variants from the sequence itself [3].

## Conclusion

Scanning for depletion of homozygous haplotypes provides a powerful tool to identify deleterious recessive alleles, especially for large genotyped livestock populations. Our results confirm the existence, relative rarity, and severe effects on fertility and welfare of lethal haplotypes in commercial livestock populations. We show that these haplotypes, apart from reduced fertility in the parent animal, also cause large numbers (several hundred at population level) of stillbirths (‘mummified piglets’), even within highly managed populations. Moreover, the method applied in this study is increasing in potential, as growing numbers of genotyped animals are becoming available for breeding purposes. Finally, this study will facilitate at least partial purging of lethal variation by avoiding matings producing affected or non-viable progeny, demonstrating its value for current breeding programs and animal welfare.

## Methods

### Animals, genotypes and pre-processing

The dataset consists of 5517 animals from a commercial synthetic boar line (cross between Large White and Piétrain), and 5301 Landrace and 12,982 Large White animals from two commercial sow lines. Three different SNP panels were used to genotype the animals for the analysis; the 10 K GeneSeek-Neogen Genomic Profiler 10 k BeadChip comprising 10,241 SNP (10 K), the Illumina Infinium PorcineSNP60 v2 BeadChip comprising 61,565 SNP (60 K), and the GeneSeek-Neogen PorcineSNP80 BeadChip comprising 68,528 SNP (80 K). An overview of the number of animals per panel is provided in Additional file 1: Table S1**.** The chromosomal positions were determined based on the *Sus scrofa* reference assembly [34]. SNPs with unknown position on Sscrofa10.2 and sex-chromosomal SNPs were discarded. Additional file 1: Table S2 provides an overview of the number of SNPs that met the following requirements: Each marker had a MAF greater than 0.01, and a call rate greater than 0.85. Only one marker was used if a genomic position contained multiple markers. Moreover animals with frequency of missing genotypes greater than 0.30 were discarded from the analysis (Additional file 1: Table S4). All pre-processing steps were performed using Plink v1.90b3.30 [35]. We did not filter for deviation of HWE because we expect the MH and DH haplotypes to deviate from HWE. The final dataset contained 22,961 animals with an average per-individual call rate of 0.987, 0.955, and 0.983 for 10 K, 60 K and 80 K SNP panels, respectively (Additional file 1: Table S3**)**. Inbreeding assessment was performed by calculating the F coefficient (observed vs expected homozygous genotype counts) in Plink v1.90b3.30 [35].

### Imputation from lower to higher density SNP panels

We used the pedigree based BEAGLE version 4.0 genetic analysis software for phasing and imputation of samples [36]. First, 10 K samples were imputed to 60 K per individual breeding line. This differs for the boar line in which we imputed 10 K samples directly to 80 K due to the smaller number of 60 K reference samples for this line. Thereafter 60 K was imputed to 80 K and one final round of phasing was performed to make full use of the family relationships (parent offspring duos and trios). Bcftools version 1.3–27-gf31e888 was used to merge vcf files [37]. Imputation accuracies are presented in Additional file 1: Table S3**.**

### Identification of missing homozygote haplotypes

We used a sliding window approach shifting along each chromosome ranging from 0.5 to 4 Mb in steps of 0.5 × window size implemented in a python module. We tested a single haplotype per locus (in case of overlapping haplotypes) with lowest *p*-value for effect on phenotypes. Haplotypes with a frequency > 0.5% were retained for identification of missing homozygotes. The expected number of homozygotes was estimated using the parental haplotype information with the formula described in Fritz et al., 2013. Moreover, the number of heterozygous offspring from carrier matings was calculated to verify whether there is a deviation from HWE. An exact binomial test was applied to test the number of observed homozygotes with the number of expected homozygotes. Haplotypes were considered significant if *P* < 5 × 10^−3^ for haplotypes with MH (0 observed) and *P* < 5 × 10^−6^ for haplotypes exhibiting DH, similar to Pausch et al. 2015 [8]. Circos software was used to visualize the haplotypes in genomic ideograms [38].

### Phenotypic effects associated with lethal haplotypes

Phenotypic records of TNB were available for all three lines. In addition, phenotypic records of NSB, and MUM were available for the two sow lines. In total, records of TNB for 4041 matings comprising 1566 sows and 432 boars were available in the boar line. Records of TNB, NSB, and MUM were available for 15,174 matings comprising 3159 sows and 1485 boars in the Landrace line and 26,961 matings comprising 6745 sows and 1671 boars in the Large White line. We examined each identified haplotype and records on TNB, NSB, and MUM are listed for all C x C matings identified in the phenotypic records. We used a Welch t-test to assess if the phenotypes from the C x C matings significantly differ from C x NC matings. A *P*-value <= 0.05 was considered significant.

### Candidate gene identification

We selected all the genes (Ensembl gene IDs) in regions of missing and deficit homozygosity to perform gene-set enrichment analysis in DAVID [39], an enrichment score > 3.0 was considered significant. Also, all porcine genes (Ensembl release 87) within the identified haplotypes were analyzed for the observed phenotypes in gene knock-out/loss-of-function studies in other mammals (mainly for early lethality). Genes that, in knock-out mice, were lethal during developing life stages (embryonic, prenatal, perinatal, neonatal, postnatal or preweaning) were marked as candidate genes [20]. Moreover further phenotypic support in other mammalian species was obtained using the OMIM database [40].

### Runs of homozygosity and extended haplotype homozygosity

The Large white population was screened for a recent selective sweep at the *BMPER* locus with an EHH test using the R package rehh [41, 42]. First, the full dataset was phased with Shapeit v2 (recommended by the rehh package) [43] with inclusion of pedigree information. EHH was generated for each SNP in both populations, identifying long and frequent haplotypes as implemented in the R package rehh [42]. The origin and footprint of selection based on haplotype structure was examined using a bifurcation diagram [41]. ROH were inferred using PLINK(v9, [35]) with at least 20 markers covering a ROH, a maximum of one heterozygous call within a stretch and minimum size of 1 Mb.

## Additional files

Additional file 1: Figure S1. F inbreeding coefficient based on observed and expected autosomal homozygous genotype counts. **Figure S2.** Genomic locations from missing (red) and depleted (blue) homozygotes in Landrace breed. **Figure S3.** Genomic locations from missing (red) and depleted (blue) homozygotes in Large white breed. **Figure S4.** Genomic locations from missing (red) and depleted (blue) homozygotes in the Boar breed. **Figure S5.** Haplotype frequency density distribution. **Figure S6.** Expected homozygotes. **Figure S7.** Litters from carrier by carrier matings for haplotype LW19 **Figure S8.** NBA/NSB/MUM piglets for LW19 C x C and C x NC matings. **Figure S9.** Example of a mummified piglet from a carrier sow inseminated with a carrier boar (C x C mating). **Table S1.** Overview of animals genotyped per panel and number of total animals in trio (both parents and offspring genotyped) in all three breeding lines. **Table S2.** Final set of markers per panel after pre-processing. **Table S3.** Imputation accuracy (BEAGLE R^2^). **Table S4.** Final set of animals after pre-processing. (PDF 1274 kb)

Additional file 2: Complete list of missing and depleted homozygotes in three commercial pig breeds. (XLSX 168 kb)

Additional file 3: Table S1. Genomic loci enriched for haplotypes exhibiting missing or depleted homozygosity. (XLSX 25 kb)

Additional file 4: Table S1. List of haplotypes affecting fertility. (XLSX 16 kb)

## Abbreviations

BR: synthetic boar line
C x C mating: Carrier by carrier mating
C x NC mating: Carrier by non-carrier mating
DH: Deficit homozygosity
EHH: Extended haplotype homozygosity
EL: Embryonic lethality
GEBV: Genomic estimated breeding value
HWE: Hardy-Weinberg equilibrium
LD: Linkage disequilibrium
LR: Landrace
LW: Large White
MAF: Minor allele frequency
MH: Missing homozygosity
MUM: Number of mummified pigs
NBA: Number born alive
NSB: Number of stillborn
ROH: Runs of homozygosity
TNB: Total number born
