# Supplementary material for: A systematic survey to identify lethal recessive variation in highly managed pig populations
Source: BMC Genomics. 2017 Nov 9;18:858. doi: 10.1186/s12864-017-4278-1 (PMC5680825; doi:10.1186/s12864-017-4278-1)
Supplement: Supplementary file 1 — F inbreeding coefficient based on observed and expected autosomal homozygous genotype counts. Figure S2. Genomic locations from missing (red) and depleted (blue) homozygotes in Landrace breed. Figure S3. Genomic locations from missing (red) and depleted (blue) homozygotes in Large white breed. Figure S4. Genomic locations from missing (red) and depleted (blue) homozygotes in the Boar breed. Figure S5. Haplotype frequency density distribution. Figure S6. Expected homozygotes. Figure S7. Litters from carrier by carrier matings for haplotype LW19 Figure S8. NBA/NSB/MUM piglets for LW19 C x C and C x NC matings. Figure S9. Example of a mummified piglet from a carrier sow inseminated with a carrier boar (C x C mating). Table S1. Overview of animals genotyped per panel and number of total animals in trio (both parents and offspring genotyped) in all three breeding lines. Table S2. Final set of markers per panel after pre-processing. Table S3. Imputation accuracy (BEAGLE R2). Table S4. Final set of animals after pre-processing. (PDF 1274 kb) [file 12864_2017_4278_MOESM1_ESM.pdf]

Figures

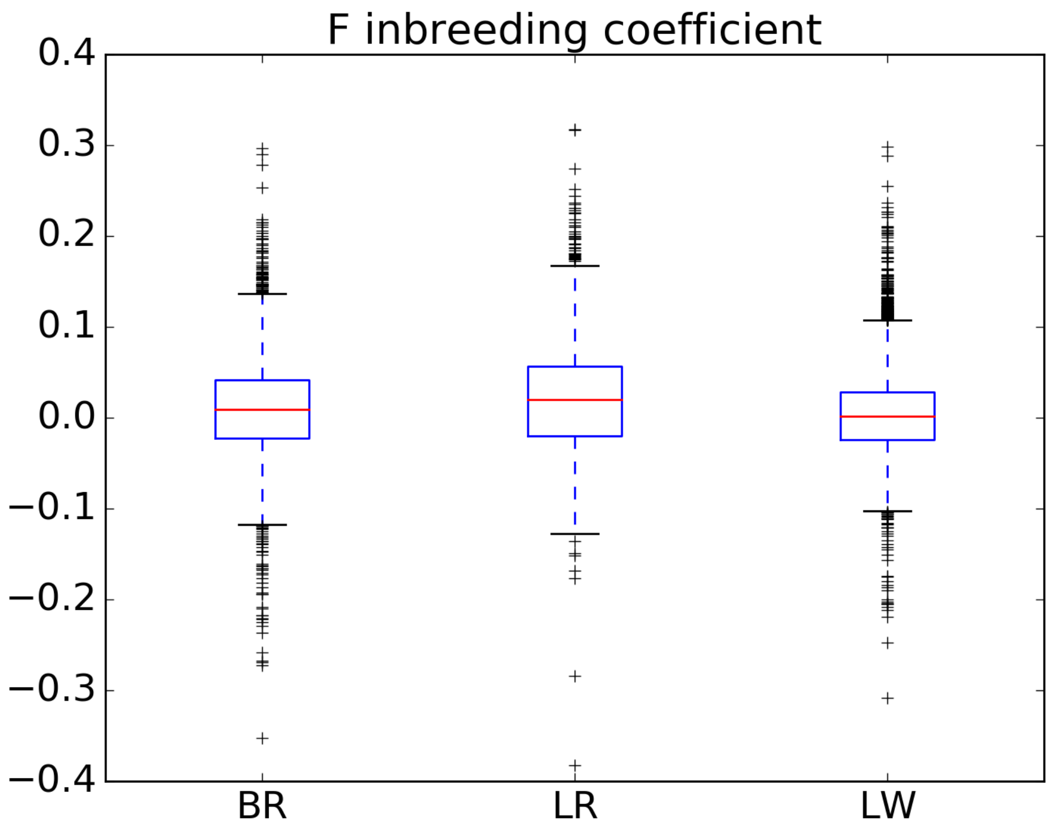

Figure S1: F inbreeding coefficient. Calculated based on observed and expected autosomal homozygous genotype counts using plink heterozygosity (--het) (Purcell et al. 2007).

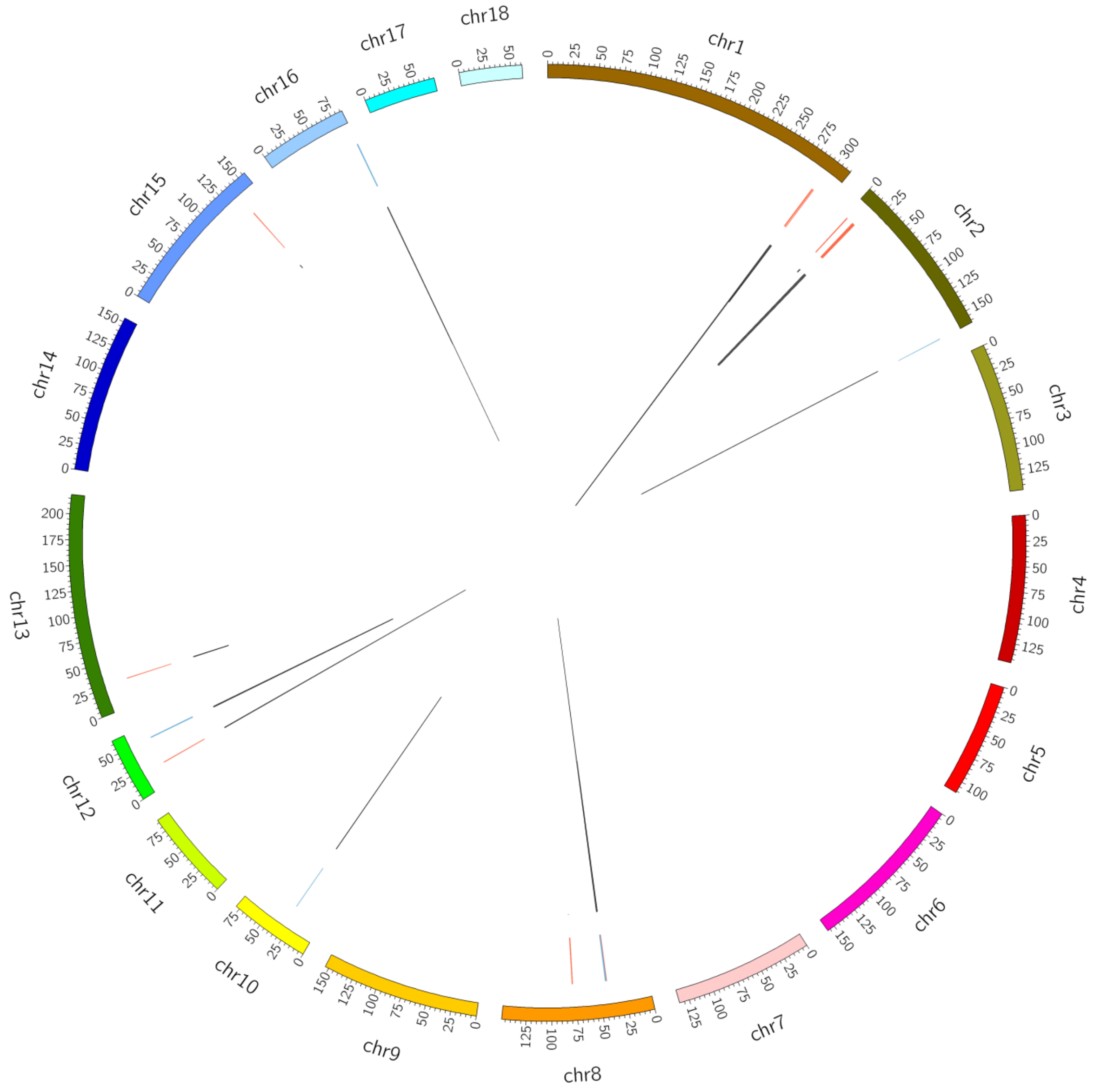

Figure S2: Genomic locations from missing (red) and depleted (blue) homozygotes in Landrace breed. Histograms show relative haplotype frequency ranging from 2.6 to 11.4%, plot created using Circos (Krzywinski et al. 2009).

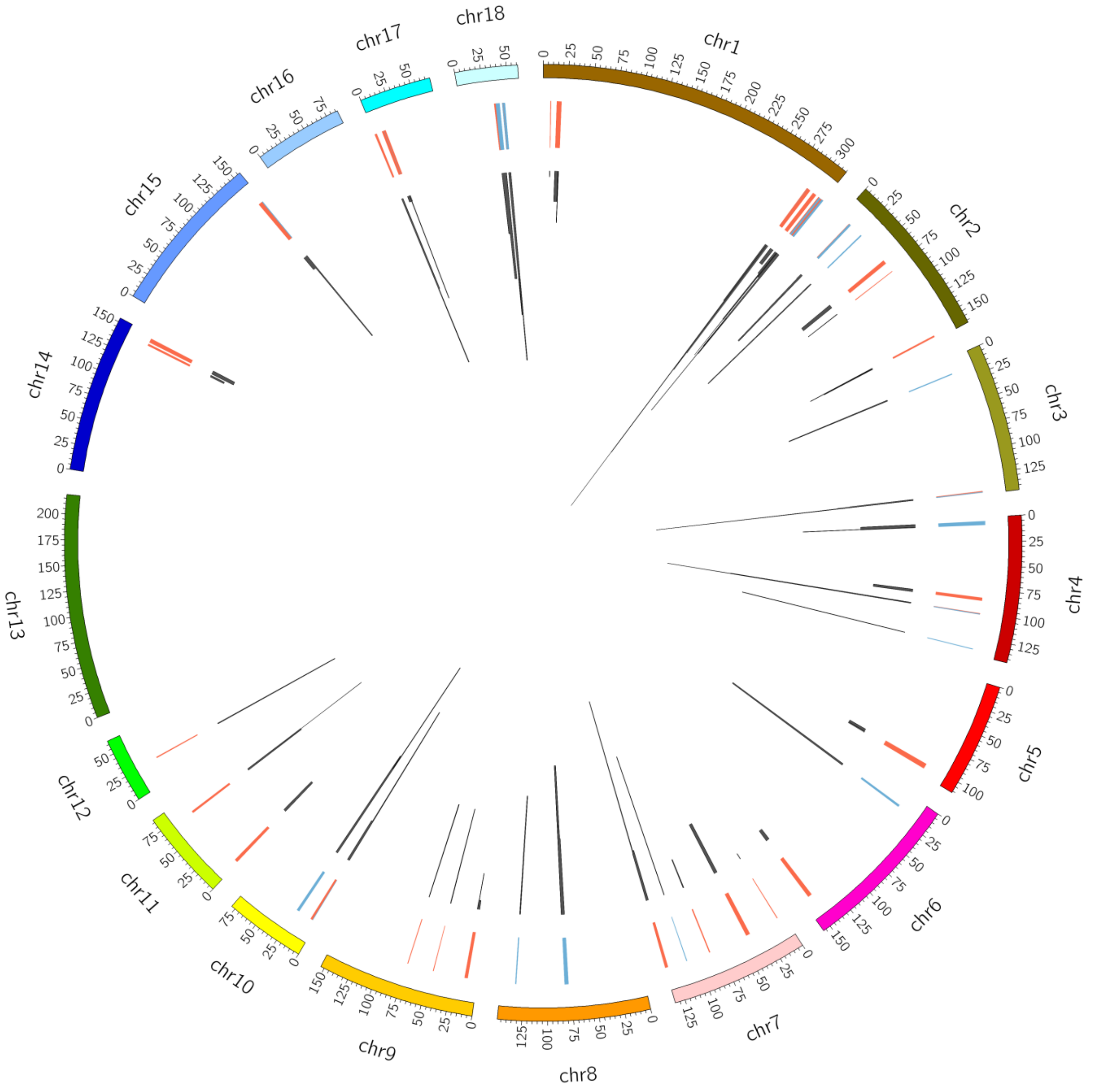

Figure S3: Genomic locations from missing (red) and depleted (blue) homozygotes in Large white breed. Histograms show relative haplotype frequency ranging from 0.8 to 11.5%, plot created using Circos (Krzywinski et al. 2009).

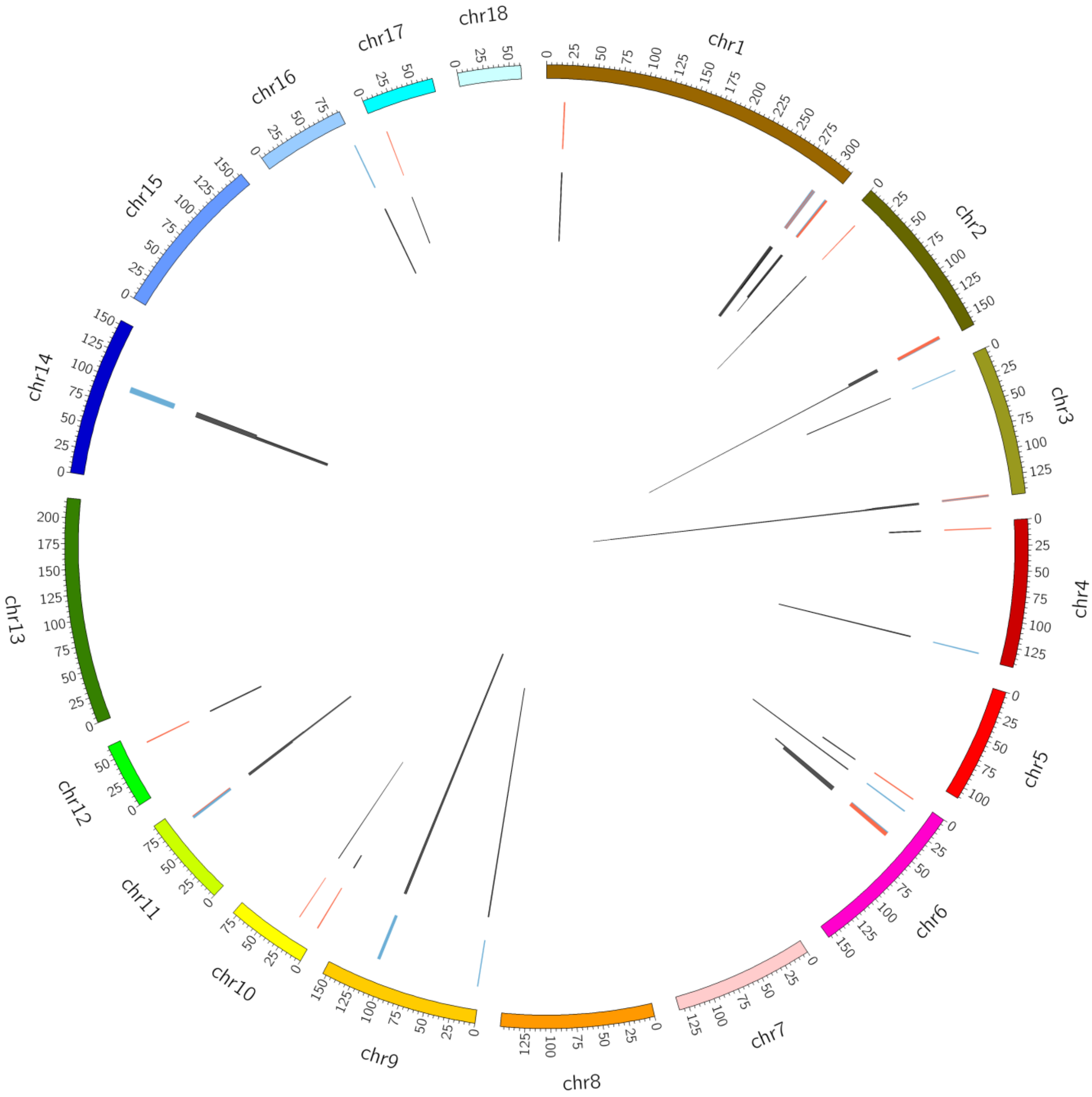

Figure S4: Genomic locations from missing (red) and depleted (blue) homozygotes in the Boar breed. Histograms show relative haplotype frequency ranging from 2.5 to 15.8%, plot created using Circos (Krzywinski et al. 2009).

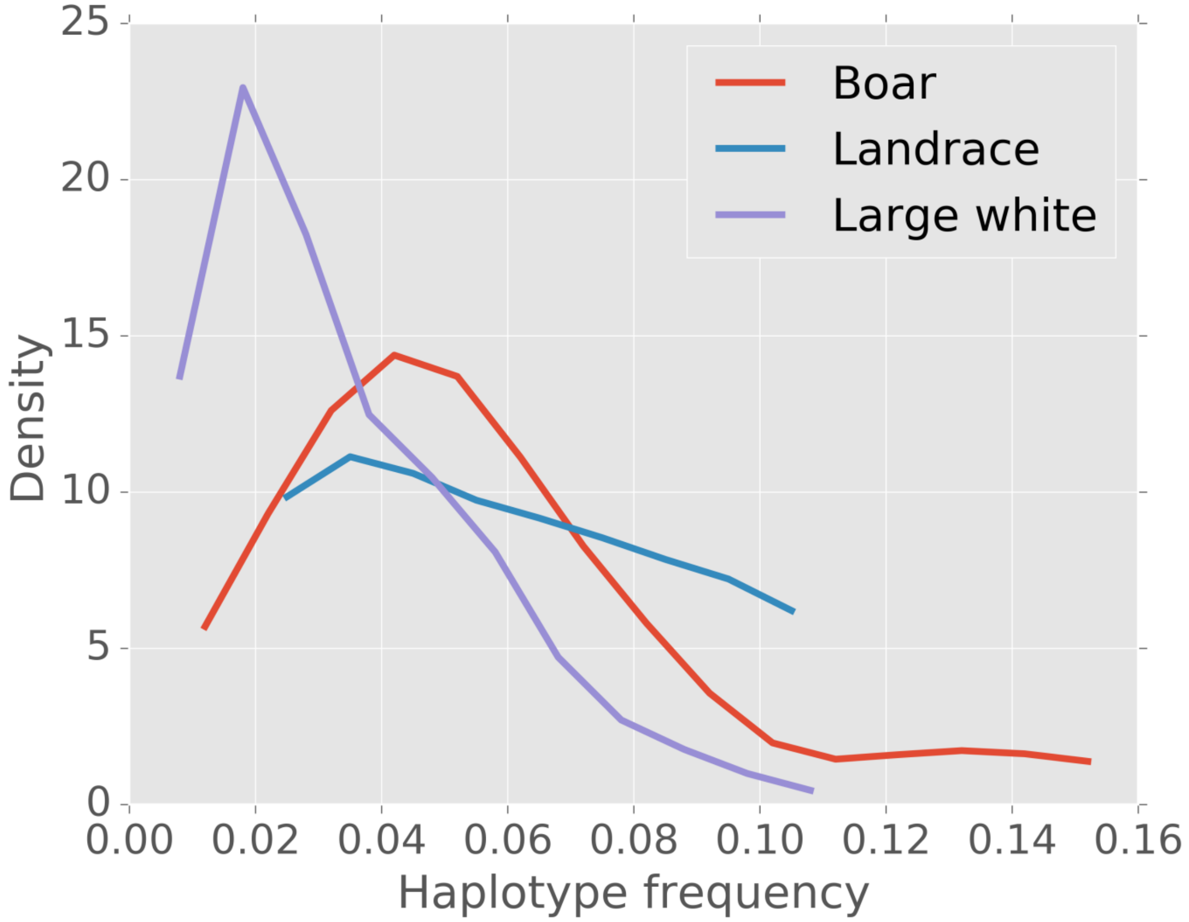

Figure S5: Haplotype frequency density distribution. The Large white breed shows higher proportion of low-frequency (<3%) haplotypes exhibiting deficit homozygosity compared to Landrace (p: 0.049) and Boar breed (p: 0.0098). Two-sample Kolmogorov-Smirnov test was used to assess significance.

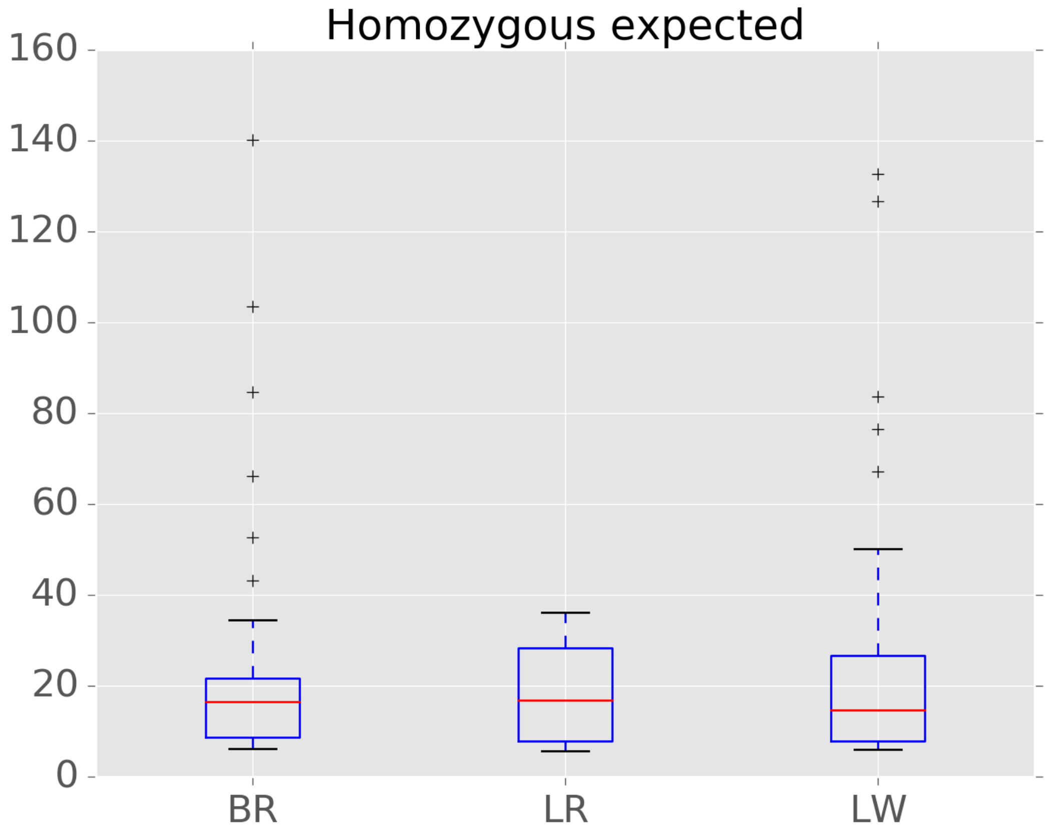

Figure S6: Expected homozygotes. Boxplot shows the number of expected homozygotes for all significant missing/deficit haplotypes identified.

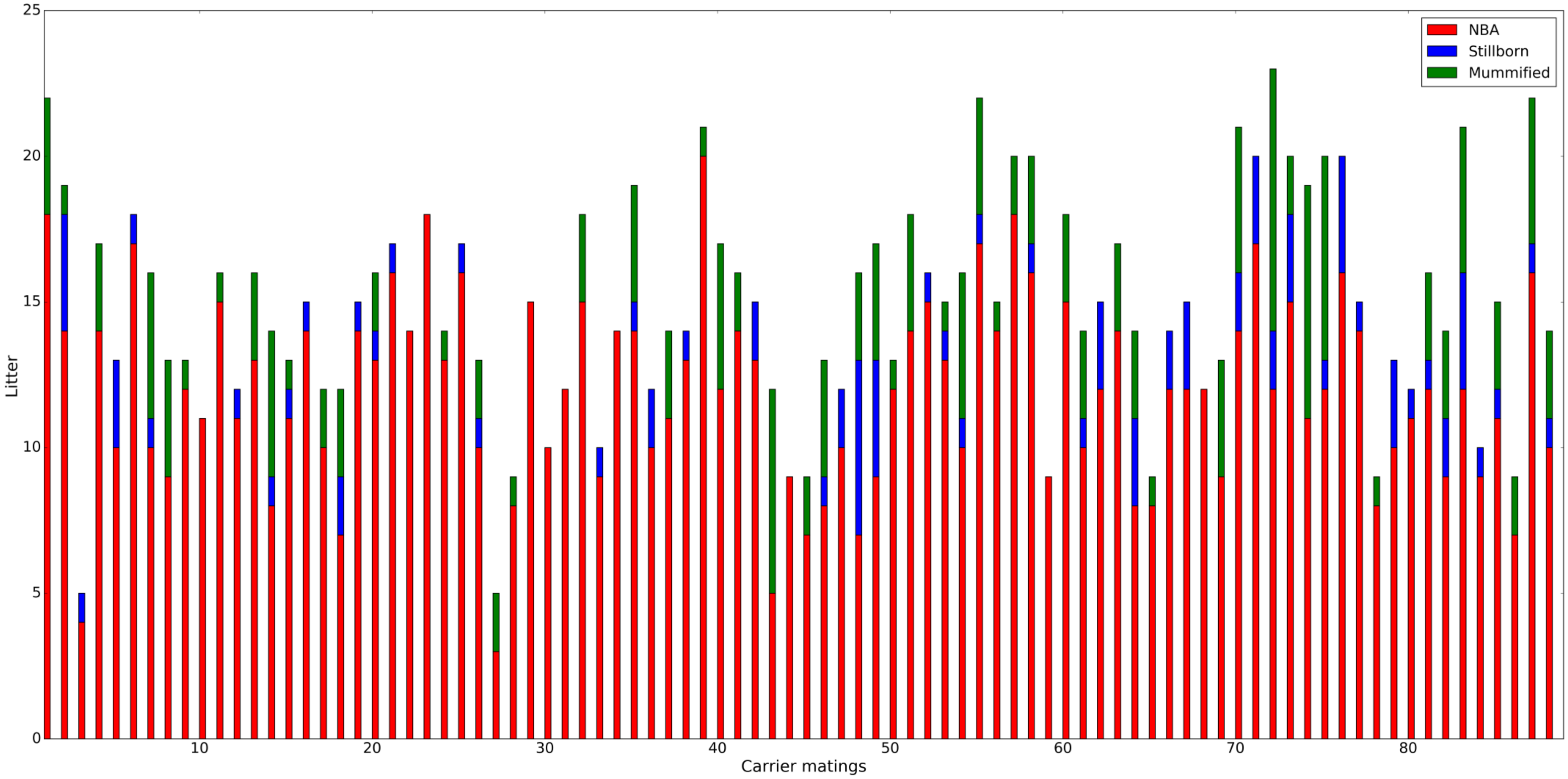

Figure S7: Litters from carrier by carrier matings for haplotype LW19. Figure shows number born alive (NBA), number of stillborn (NSB), and number of mummified piglets (MUM) for 88 litters.

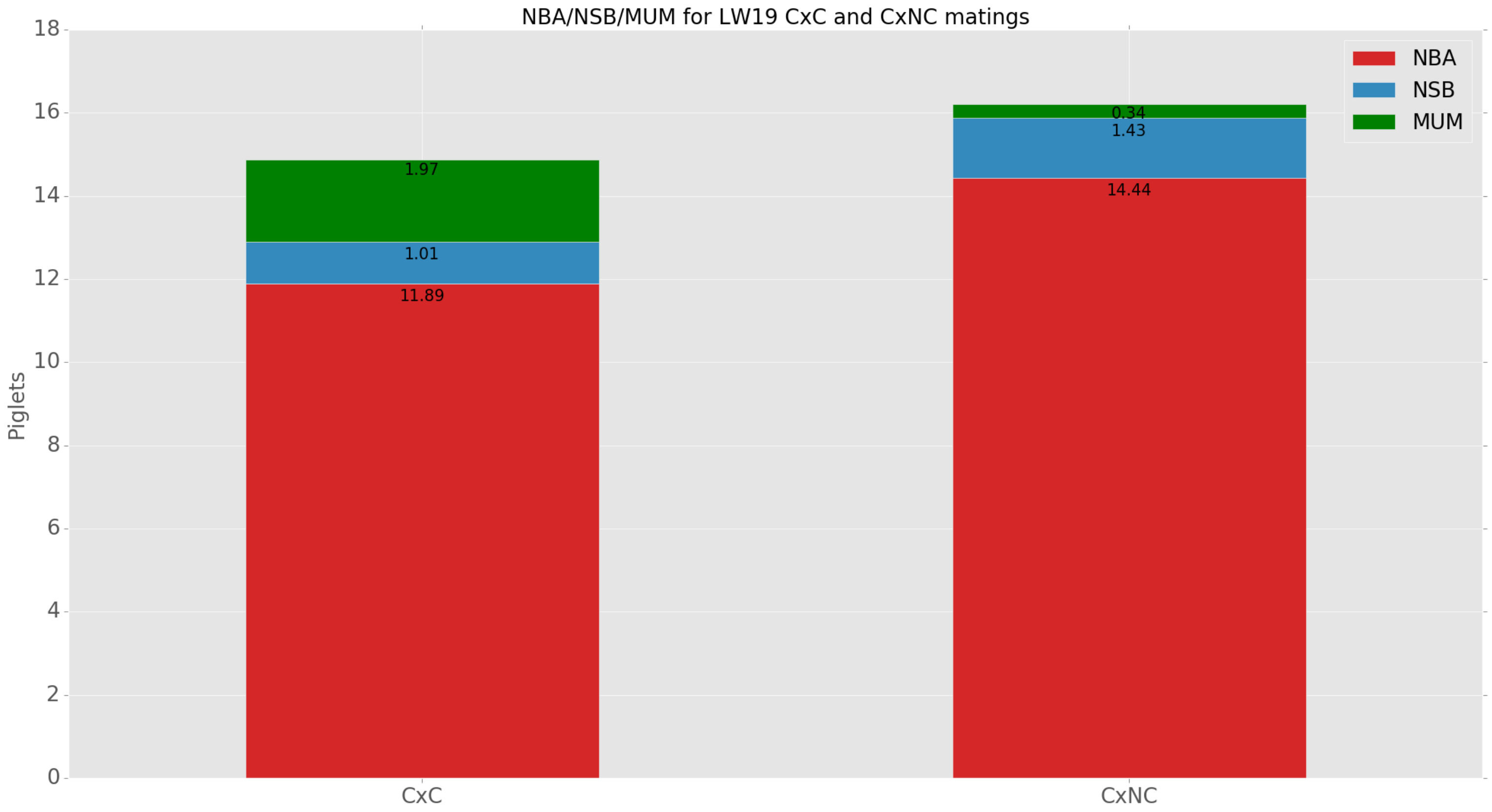

Figure S8: NBA/NSB/MUM piglets for LW19 CxC and CxNC matings. Figure shows that the loss in NBA for CxC matings coincides with an increase in MUM. The average ratio MUM/TNB for the C x C matings is 15.3% (1.97 / 12.9) compared to only 2.14% (0.34 / 15.87) of C x NC matings.

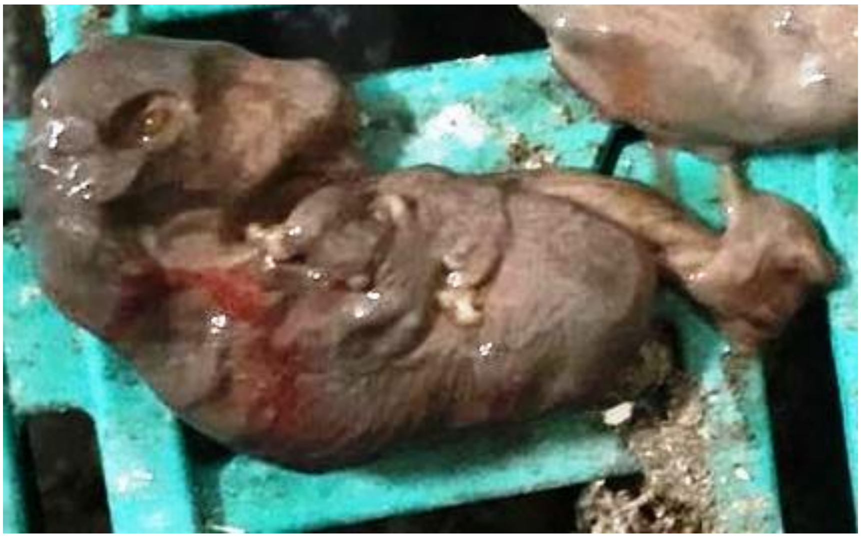

Figure S9: Example of a mummified piglet from a carrier sow inseminated with a carrier boar (C x C mating). The sow farrowed on the 11th of May 2017 on a farm in the Netherlands. The litter consists of twelve live-born piglets and three mummified piglets.

Tables

Table S1: Overview of animals genotyped per panel and number of total animals in trio (both parents and offspring genotyped) in all three breeding lines.

Line	# 10K	#60K	#80K	#Total (Trio)
**BR**	1,904	846	2,767	5,517 (3,806)
**LR**	1,047	2,699	1,555	5,301 (2,548)
**LW**	4,035	3,305	5,642	12,982 (8,778)
**Total**	6,986	6,850	9,964	23,800 (15,132)

Table S2: Final set of markers per panel after pre-processing.

Line	# 10K	#60K	#80K
BR	7,245	35,854	45,429
LR	7,719	38,699	48,695
LW	7,613	37,329	47,590

Table S3: Imputation accuracy (BEAGLE R^2 (Browning and Browning 2007)).

Line	60K phasing	80K phasing	10K > 60K imputation*	60K > 80K imputation	10K - 60K -80K phasing	WGS imputation
BR	0.9990	0.9979	0.9563	0.9741	0.9960	0.9593
LR	0.9986	0.9993	0.9474	0.9784	0.9983	0.9608
LW	0.9995	0.9995	0.9574	0.9836	0.9985	0.9401

*(80K for BR line)

Table S4: Final set of animals after pre-processing. Average call rate is presented between parentheses.

Line	# 10K	#60K	#80K	#Total
BR	1,904 (0.987)	824 (0.919)	2,760 (0.983)	5,488
LR	1,047 (0.987)	2,459 (0.962)	1,550 (0.984)	5,056
LW	4,035 (0.987)	2,746 (0.957)	5,636 (0.983)	12,417
Total	6,986 (0.987)	6,029 (0.955)	9,964 (0.983)	22,961

Table S5: DAVID (Huang et al. 2007) gene-set enrichment analysis annotation cluster 1 (Enrichment Score: 3.45).

Category	Term	Count	%	PValue	List Total	Pop Hits	Pop Total	Fold Enrichment	Benjamini
GOTERM_MF_DIRECT	GO:0005549: odorant binding	19	2.32	5.02E-08	694	92	16313	4.85	4.14E-05
KEGG_PATHWAY	hsa04740: Olfactory transduction	37	4.51	2.06E-05	302	399	6910	2.12	0.005025
INTERPRO	IPR000276: G protein-coupled receptor, rhodopsin-like	51	6.22	2.87E-04	774	721	18593	1.70	0.289133
UP_KEYWORDS	Olfaction	34	4.15	4.50E-04	820	443	20568	1.93	0.046184
INTERPRO	IPR017452: GPCR, rhodopsin-like, 7TM	51	6.22	4.86E-04	774	738	18593	1.66	0.250727
INTERPRO	IPR000725: Olfactory receptor	34	4.15	5.55E-04	774	430	18593	1.90	0.19749
GOTERM_BP_DIRECT	GO:0050911: Detection of chemical stimulus involved in sensory perception of smell	34	4.15	6.54E-04	715	425	16787	1.88	0.845323
GOTERM_MF_DIRECT	GO:0004984 Olfactory receptor activity	34	4.15	8.21E-04	694	431	16313	1.85	0.287518
UP_KEYWORDS	Transducer	56	6.83	0.001095	820	901	20568	1.56	0.073798
UP_KEYWORDS	G-protein coupled receptor	53	6.46	0.001252	820	844	20568	1.58	0.072384
UP_KEYWORDS	Sensory transduction	40	4.88	0.002588	820	610	20568	1.64	0.127217
GOTERM_BP_DIRECT	GO:0007186: G-protein coupled receptor signaling pathway	56	6.83	0.004255	715	899	16787	1.46	0.997707
GOTERM_MF_DIRECT	GO:0004930: G-protein coupled receptor activity	46	5.61	0.005148	694	713	16313	1.52	0.655101

References

Browning SR, Browning BL. 2007. Rapid and accurate haplotype phasing and missing-data inference for whole-genome association studies by use of localized haplotype clustering. *Am J Hum Genet* **81**(5): 1084-1097.

Huang DW, Sherman BT, Tan Q, Collins JR, Alvord WG, Roayaei J, Stephens R, Baseler MW, Lane HC, Lempicki RA. 2007. The DAVID Gene Functional Classification Tool: a novel biological module-centric algorithm to functionally analyze large gene lists. *Genome Biol* **8**(9).

Krzywinski M, Schein J, Birol I, Connors J, Gascoyne R, Horsman D, Jones SJ, Marra MA. 2009. Circos: an information aesthetic for comparative genomics. *Genome research* **19**(9): 1639-1645.

Purcell S, Neale B, Todd-Brown K, Thomas L, Ferreira MAR, Bender D, Maller J, Sklar P, de Bakker PIW, Daly MJ et al. 2007. PLINK: A tool set for whole-genome association and population-based linkage analyses. *Am J Hum Genet* **81**(3): 559-575.
